# Nosocomial infections due to multidrug-resistant bacteria in cancer patients: a six-year retrospective study of an oncology Center in Western China

**DOI:** 10.1186/s12879-020-05181-6

**Published:** 2020-06-29

**Authors:** Ai-Min Jiang, Xin Shi, Na Liu, Huan Gao, Meng-Di Ren, Xiao-Qiang Zheng, Xiao Fu, Xuan Liang, Zhi-Ping Ruan, Yu Yao, Tao Tian

**Affiliations:** 1 Department of Medical Oncology, The First Affiliated Hospital of Xi’an Jiaotong University, No. 277 Yanta West Road, Xi’an, Shaanxi 710061 People’s Republic of China; 2 School of Public Health, Xi’an Jiaotong University Health Science Center, No. 277 Yanta West Road, Xi’an, Shaanxi 710061 People’s Republic of China

**Keywords:** Cancer patients, Nosocomial infections, Multidrug-resistant bacteria, Risk factors

## Abstract

**Background:**

Bacterial infections are the most frequent complications in patients with malignancy, and the epidemiology of nosocomial infections among cancer patients has changed over time. This study aimed to evaluate the characteristics, antibiotic resistance patterns, and prognosis of nosocomial infections due to multidrug-resistant (MDR) bacteria in cancer patients.

**Methods:**

This retrospective observational study analyzed cancer patients with nosocomial infections caused by MDR from August 2013 to May 2019. The extracted clinical data were recorded in a standardized form and compared based on the survival status of the patients after infection and during hospitalization. The data were analyzed using independent samples t-test, Chi-square test, and binary logistic regression. *P*-values < 0.05 were considered significant.

**Results:**

One thousand eight patients developed nosocomial infections during hospitalization, with MDR strains detected in 257 patients. Urinary tract infection (38.1%), respiratory tract infection (26.8%), and bloodstream infection (BSI) (12.5%) were the most common infection types. Extended-spectrum β-lactamase producing Enterobacteriaceae (ESBL-PE) (72.8%) members were the most frequently isolated MDR strains, followed by *Acinetobacter baumannii* (11.7%), and *Stenotrophomonas maltophilia* (6.2%). The results of multivariate regression analysis revealed that smoking history, intrapleural/abdominal infusion history within 30 days, the presence of an indwelling urinary catheter, length of hospitalization, and hemoglobin were independent factors for in-hospital mortality in the study population. The isolated MDR bacteria exhibited high rates of sensitivity to amikacin, meropenem, and imipenem.

**Conclusions:**

The burden of nosocomial infections due to MDR bacteria is considerably high in oncological patients, with ESBL-PE being the most predominant causative pathogen. Our findings suggest that amikacin and carbapenems actively against more than 89.7% of MDR isolates. The precise management of MDR bacterial infections in cancer patients may improve the prognosis of these individuals.

## Background

Bacterial infections are the most frequent complications in patients with malignancy as these patients are more likely to be immunocompromised due to malnutrition, invasive procedures, surgery, chemotherapy, radiation, and some new treatment modalities [1, 2]. There is growing evidence suggesting that infection in cancer patients is associated with a delayed initiation of chemotherapy, reduced standard dosage, prolonged hospitalization, increased financial burden of healthcare, and more severe morbidity and mortality [3–5]. The epidemiology of nosocomial infections among cancer patients has changed over time, and the causative organisms of nosocomial infections have shifted from gram-positive pathogens to gram-negative pathogens in the last 20 years worldwide [6, 7].

Most of the previous studies [8–11] showed that extended-spectrum β-lactamase-producing Enterobacteriaceae (ESBL-PE), multidrug-resistant (MDR) *Pseudomonas aeruginosa*, carbapenem-resistant Enterobacteriaceae (CRE), *Acinetobacter baumannii* and methicillin-resistant *Staphylococcus aureus* (MRSA) have been increasingly identified as the predominant causative pathogens in cancer patients due to the phenomenon of antibiotics misuse [12, 13]. To the best of our knowledge, previously published guidelines recommend antibiotic treatment for cancer patients with neutropenia or septic shock [14–16]. However, no explicit empirical antibiotic therapy regimens were recommended for highly suspected MDR bacteria causing nosocomial infections in cancer patients. In addition, only limited data are available for the bacterial distribution, antibiotic resistance patterns, and prognosis of these infections in oncological patients. Therefore, we conducted this retrospective study to explore the clinical characteristics, microbial spectrum, antibiotic resistance patterns, and prognostic factors of nosocomial infections due to MDR bacteria in cancer patients hospitalized in the First Affiliated Hospital of Xi’an Jiaotong University from August 2013 to June 2019 to provide evidence for clinical practice.

## Methods

### Study population and design

We conducted a single-center retrospective observational study in a 2560-bed university referral cancer center in Xi’an, China, enrolling cancer patients who received medical care during hospitalization from August 2013 to May 2019. The electronic medical record database was reviewed to identify cancer patients with nosocomial infections due to MDR bacteria. All cancer patients diagnosed with nosocomial infections below the age of 18 or without complete medical records were excluded, and only the initial infection episode was analyzed.

### Data collection

Data in electronic medical records of all included cancer patients were extracted. The extracted clinical data included age, gender, smoking history, Eastern Cooperative Oncology Group (ECOG) performance status, primary location of the disease, existence of distant metastasis, American Joint Committee on Cancer (AJCC) TNM categories, primary sites of infection, comorbidities and severity of underlying conditions according to the Charlson comorbidity index (CCI) [17], existence of fever, types of cancer therapy within 30 days (surgery, chemotherapy, radiotherapy, or concurrent chemoradiotherapy), corticosteroid treatment within previous 30 days, prior infection before hospital admission, granulocyte colony-stimulating factor (G-CSF) use within 30 days, previous antibiotics treatment within 30 days, the presence of indwelling catheters or other devices, invasive procedure within previous 30 days, the types of microbiological samples, empirical antibiotics treatment, effective empirical antibiotics treatment, length of antibiotics treatment, intensive care unit (ICU) admission during hospitalization, existence of septic shock, mechanical ventilation, outcome of the analyzed infection episode during hospitalization (death or discharged), the worst values of laboratory parameters before infection diagnosis including blood routine test, serum albumin, procalcitonin (PCT), and antibiotic susceptibility tests of isolated pathogens.

### Definitions

MDR infection episodes were defined by our physicians in the following situations: (1) patients had at least one positive test for a MDR pathogen clinical sample (such as ESBL-PE, AmpC cephalosporinase hyperproducing Enterobacteriaceae, β-lactamase OXA-type-producing Enterobacteriaceae, *Stenotrophomonas maltophilia*, MDR *Pseudomonas aeruginosa* and *Acinetobacter baumannii*, vancomycin-resistant *Enterococcus faecium*, and MRSA [18]); (2) clinical symptoms and signs, laboratory, or radiology examination results indicated infection according to the descriptions of the Centers for Disease Control and Prevention [19]; or (3) there was a clear infection diagnosis in the patient’s electronic medical charts.

MDR gram-negative bacteria (MDRGNB) were defined as gram-negative bacteria exhibiting resistance to at least one agent in each of three or more categories of antimicrobial agents, including β-lactam/β-lactamase inhibitor combinations (piperacillin/tazobactam), extended-spectrum cephalosporins (ceftriaxone, ceftazidime, cefepime), carbapenems (imipenem/meropenem), monobactams, aminoglycosides (gentamicin, amikacin) and/or fluoroquinolones, while MDR gram-positive bacteria were defined as gram-positive bacteria exhibiting resistance to vancomycin for *Enterococcus faecium*, and resistant to methicillin for *Staphylococcus aureus* [14]. Clinical samples such as sputum, urine, blood culture, stool, wounds secreta, ascites, pleural, drainage fluid postoperation, and other samples were collected once patients were suspected of MDR infection. The swabs were collected for colonization screening but not for the diagnosis of MDR infection episodes.

The antimicrobial susceptibility of isolated organisms was determined using the Kirby-Bauer disk diffusion method according to the Clinical and Laboratory Standards Institute (CLSI) guidelines [20]. ESBL-resistant organisms were defined as being resistant to one or more extended-spectrum cephalosporins, and ESBL producers were confirmed via the double-disk synergy test.

Nosocomial infection was defined as signs or symptoms of infection that occurred > 48 h after hospital admission or < 48 h after hospital discharge. Otherwise, the case was considered community-onset [21].

Fever was considered as an axillary temperature of 38.3 °C on one occasion or a temperature of > 38.0 °C on two or more occasions during 12 h [22].

Empirical antibiotics treatment was considered as the initiation of antimicrobial agents before the results of microbiology and communicated to clinicians [13]. Empirical antibiotics treatment was considered effective once the antibiotics used could suppress the activity of the isolated MDR pathogens according to the results of antimicrobial susceptibility tests [13].

### Study outcomes

The present study aimed to describe the clinical characteristics, microbial spectrum, antibiotic resistance patterns, and prognostic factors of all cancer patients with nosocomial infections caused by MDR bacteria and to determine the in-hospital mortality and its associated risk factors. In-hospital mortality was defined as death during hospitalization for the studied infection episodes.

### Statistical analysis

The extracted clinical data were recorded in a standardized form and compared based on the patient’s survival status after infection during hospitalization. Parametric continuous quantitative variables are reported as the means and standard deviation, while medians and interquartile ranges were used for nonparametric continuous variables. Continuous variables were analyzed by the independent samples t-test or the Mann-Whitney U test. Categorical variables were analyzed by the chi-square or Fisher’s exact tests. Univariate and multivariate logistic regression analyses were used to investigate risk factors for in-hospital mortality of cancer patients with nosocomial infections caused by MDR bacteria. Variables with a *p*-value of < 0.10 from univariate analysis and variables with clinical significance were included in a multivariate logistic regression analysis using stepwise selection. All statistical analyses were performed using the SPSS version 22.0 for Windows (SPSS Inc., Chicago, IL, USA).

## Results

### Essential characteristics of the study population

**Fig. 1:**
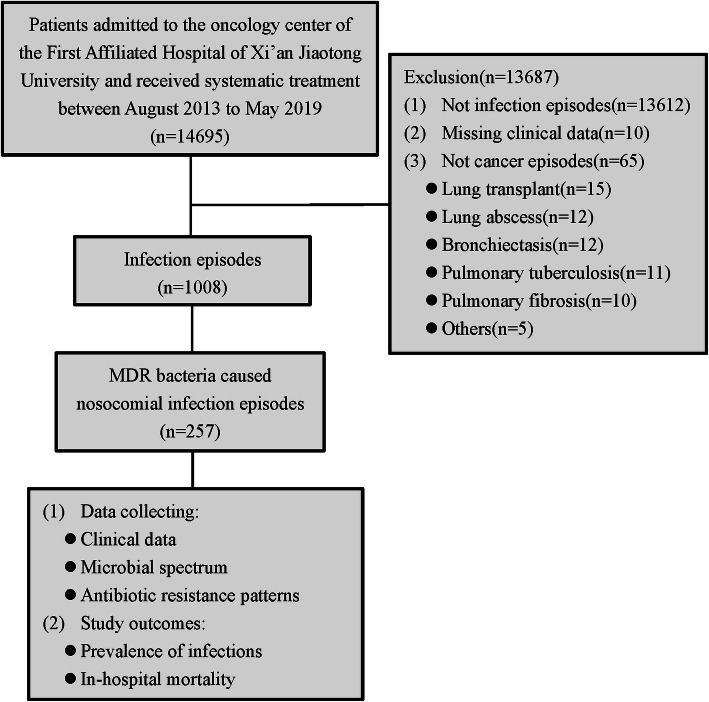
Flow chart of the study

During the six years, there were 14,695 patients admitted to the oncology center of the First Affiliated Hospital of Xi’an Jiaotong University who received systemic treatment. In total, 1008 cancer patients developed nosocomial infections, and MDR strains were detected in 257 cases (Fig. 1). Among the study subjects, there were 117 (45.5%) males and 140 (54.5%) females, with an average age of 59.6 ± 11.5 years. Diabetes mellitus, renal disease, and liver disease was observed in 20 (7.8%), 15 (5.8%), and 11 (4.3%) of patients, respectively. The most frequent diagnoses were esophago-gastrointestinal cancer, gynecological cancer, colon and rectal cancer, and breast cancer, which were observed in 74 (28.8%), 63 (24.5%), 34 (13.2%) and 21 (8.2%) of cases, respectively. Overall, 183 (71.2%) of patients had a performance status greater than 2, 66 (25.7%) had distant metastasis, 109 (42.4%) had received surgery within 30 days, 90 (35.0%) had received chemotherapy within 30 days, 34 (13.2%) had received radiotherapy within 30 days, and 37 (14.4%) had received concurrent chemoradiotherapy within 30 days. Eighty-nine (34.6%) patients had a drain postoperation, 86 (33.5%) had an indwelling urinary catheter, and 67 (26.1%) had a nasogastric tube (Table 1).

**Table 1:** Clinical characteristics of cancer patients who survived or died during hospitalization of nosocomial infections caused by MDR bacteria

Characteristics	All *N* = 257	Univariate analysis			Multivariate analysis	
		Survivor *N* = 229, n (%)	Non-survivor *N* = 28, n (%)	*P*-value	OR (95% CI)	*P*-value
**Demographic data**						
Sex (male)	117 (45.5)	103 (45.0)	14 (50.0)	0.614		
Age (years)	59.6 ± 11.5	59.1 ± 11.4	63.6 ± 12.2	0.052	1.03 (0.96–1.10)	0.413
Smoking history				0.039*		0.048*
Never smoker	171 (66.5)	153 (66.8)	18 (64.3)		REF (1.00)	
Former smoker	46 (17.9)	37 (16.2)	9 (32.1)		8.38 (1.10–63.74)	0.040*
Current smoker	40 (15.6)	39 (17.0)	1 (3.6)		0.44 (0.03–7.25)	0.373
**ECOG performance status**				0.002*		
0,1	183 (71.2)	170 (74.2)	13 (46.4)		REF (1.00)	
2,3,4	74 (28.8)	59 (25.8)	15 (53.6)		2.91 (0.55–15.34)	0.207
**Underlying cancer type**						
Head and neck cancer	4 (1.6)	3 (1.3)	1 (3.6)	0.371		
Lung cancer	19 (7.4)	17 (7.4)	2 (7.1)	1.000		
Esophago-gastrointestinal cancer	74 (28.8)	65 (28.4)	9 (32.1)	0.678		
Colon and rectal cancer	34 (13.2)	30 (13.1)	4 (14.3)	1.000		
Hepatobiliary and pancreatic cancer	10 (3.9)	8 (3.5)	2 (7.1)	0.671		
Breast cancer	21 (8.2)	18 (7.9)	3 (10.7)	0.877		
Genitourinary cancer	15 (5.8)	14 (6.1)	1 (3.6)	0.909		
Gynecological cancer	63 (24.5)	59 (25.8)	4 (14.3)	0.183		
Lymphoma	4 (1.6)	3 (1.3)	1 (3.6)	0.371		
Others^a^	13 (5.1)	12 (5.2)	1 (3.6)	1.000		
**Existence of distant metastasis**				0.008*	1.32 (0.19–9.33)	0.778
None	191 (74.3)	176 (76.9)	15 (53.6)			
Yes	66 (25.7)	53 (23.1)	13 (46.4)			
**Stage of cancer**				0.143		
Stage I	48 (18.7)	44 (19.2)	4 (14.3)			
Stage II	76 (29.6)	71 (31.0)	5 (17.9)			
Stage III	60 (23.3)	54 (23.6)	6 (21.4)			
Stage IV	73 (28.4)	60 (26.2)	13 (46.4)			
**Comorbidities**						
Cerebrovascular disease	6 (2.3)	4 (1.7)	2 (7.1)	0.130		
Liver disease	11 (4.3)	7 (3.1)	4 (14.3)	0.023*	7.17 (0.33–154.00)	0.208
Diabetes	20 (7.8)	17 (7.4)	3 (10.7)	0.810		
Renal disease	15 (5.8)	11 (4.8)	4 (14.3)	0.111		
**CCI**				0.005*		0.917
0	203 (79.0)	187 (81.7)	16 (57.1)		REF (1.00)	
1–2	49 (19.1)	39 (17.0)	10 (35.7)		0.73 (0.14–3.77)	0.709
≥ 3	5 (1.9)	3 (1.3)	2 (7.1)		1.08 (0.02–58.19)	0.968
**Existence of fever**	103 (40.1)	88 (38.4)	15 (53.6)	0.123		
**Surgery (within 30 days)**				0.003*		0.066
None	148 (57.6)	124 (54.1)	24 (85.7)		REF (1.00)	
Curative surgery	93 (36.2)	91 (39.7)	2 (7.1)		0.01 (0.00–0.53)	0.024
Palliative surgery	16 (6.2)	14 (6.1)	2 (7.1)		0.36 (0.02–6.02)	0. 479
**Chemotherapy (within 30 days)**				0.026*		0.800
None	167 (65.0)	146 (63.8)	21 (75.0)		REF (1.00)	
Neoadjuvant	1 (0.4)	1 (0.4)	0		0	1.000
Adjuvant	57 (22.2)	54 (23.6)	3 (10.7)		0	0.998
1st line	20 (7.8)	20 (8.7)	0		3.11 (0.22–44.84)	0.405
2nd line	7 (2.7)	4 (1.7)	3 (10.7)		0.05 (0.00–12.95)	0.285
≥ 3rd line	5 (1.9)	4 (1.7)	1 (3.6)		0.74 (0.08–7.02)	0.796
**Radiotherapy (within 30 days)**	34 (13.2)	31 (13.5)	3 (10.7)	0.904		
**Concurrent chemoradiotherapy** **(within 30 days)**	37 (14.4)	36 (15.7)	1 (3.6)	0.149		
**Intrapleural/abdominal infusion** **(within 30 days)**	10 (3.9)	5 (2.2)	5 (17.9)	< 0.001*	23.92 (2.16–264.29)	0.010*
**Corticosteroid therapy (within 30 days)**	125 (48.6)	112 (48.9)	13 (46.4)	0.804		
**Prior infection (within 30 days)**	13 (5.1)	10 (4.4)	3 (10.7)	0.322		
**Prior G-CSF use (within 30 days)**	90 (35.0)	84 (36.7)	6 (21.4)	0.110		
**Prior antibiotics use (within 30 days)**	12 (4.7)	11 (4.8)	1 (3.6)	1.000		
**Presence of indwelling catheters** **or other devices**						
Biliary stent	2 (0.8)	2 (0.9)	0	1.000		
Ureteral stent	15 (5.8)	15 (6.6)	0	0.333		
Indwelling urinary catheters	86 (33.5)	82 (35.8)	4 (14.3)	0.023*	31.62 (1.28–262.79)	0.035*
CVC (port-a-cath or PICC)	35 (13.6)	31 (13.5)	4 (14.3)	1.000		
Percutaneous pleural drainage tube	55 (21.4)	51 (22.3)	4 (14.3)	0.331		
Percutaneous abdomen drainage tube	6 (2.3)	4 (1.7)	2 (7.1)	0.130		
Drains postoperation	89 (34.6)	85 (37.1)	4 (14.3)	0.017*	1.43 (0.06–37.43)	0.829
Nasogastric tube	67 (26.1)	62 (27.1)	5 (17.9)	0.294		
**Invasive procedure (within 30 days)**	157 (61.1)	142 (62.0)	15 (53.6)	0.387		

*Abbreviations*: *MDR* multidrug-resistant, *OR* odds ratio, *CI* confidence interval, *ECOG* Eastern Cooperative Oncology Group, *CCI* Charlson Co-morbidity Index score, *G-CSF* granulocyte colony-stimulating factor, *CVC* central venous catheter, *PICC* peripherally inserted central catheter

^a^ Others: primitive neuroectodermal tumor (4 patients), duodenal carcinoma three patients, thymic carcinoma, carcinoid cancer of appendix, and sarcoma two patients each

“*” represents *P* < 0.05

### Infection-related data of cancer patients with nosocomial infections due to MDR bacteria

We reviewed all of the clinical data for nosocomial infections caused by MDR bacteria in cancer patients. In our study, approximately 13 (5.1%) of patients had a prior infection history within 30 days, and 12(4.7%) patients had received antibiotics therapy within 30 days. Urinary tract infection was the leading cause of nosocomial infections, accounting for 38.1% of cases, followed by respiratory tract infection (26.8%) and BSI (12.5%). There were 224 (87.2%) patients who received empirical antibiotics treatment during hospitalization. Of these patients, 48.6% of patients received combination therapy, followed by β-lactam/β-lactamase inhibitor combinations (16.0%), cephalosporins (8.6%), and fluoroquinolones (8.6%). Among patients who received empirical antibiotics therapy, the treatment was considered effective for 134 patients. The median length of antibiotics treatment for the infection episodes was 8.0 days (range 5.0–13.0), and the median duration of hospitalization was 21.0 days (range 12.0–28.0). In addition, there were 33 (12.8%) patients admitted to the ICU, 18 (7.0%) patients received mechanical ventilation, and 38 (14.8%) patients experienced septic shock (Table 2).

**Table 2:** Infection-related characteristics of cancer patients who survived or died during hospitalization of nosocomial infections caused by MDR bacteria

Characteristics	All *N* = 257	Univariate analysis			Multivariate analysis	
		Survivor *N* = 229, n (%)	Non-survivor *N* = 28, n (%)	*P*-value	OR (95% CI)	*P*-value
**Sample type**						
Sputum	62 (24.1)	53 (23.1)	9 (32.1)	0.293		
Urine	93 (36.2)	91 (39.7)	2 (7.1)	0.001*	0.01 (0–1.10)	0.054
Blood culture	44 (17.1)	32 (14.0)	12 (42.9)	< 0.001*	1.21 (0.03–50.21)	0.919
Ascites	10 (3.9)	9 (3.9)	1 (3.6)	1.000		
Wounds secreta	21 (8.2)	20 (8.7)	1 (3.6)	0.565		
Bronchoalveolar lavage fluid	6 (2.3)	4 (1.7)	2 (7.1)	0.262		
Drainage fluid post-operation	21 (8.2)	20 (8.7)	1 (3.6)	0.565		
**Primary sites of infection**						
Respiratory tract	69 (26.8)	56 (24.5)	13 (46.4)	0.013*	5.36 (0.43–66.55)	0.191
Urinary tract	98 (38.1)	94 (41.0)	4 (14.3)	0.006*	0.58 (0.04–8.21)	0.686
Skin and soft tissue	19 (7.4)	18 (7.9)	1 (3.6)	0.663		
Thoracic cavity	13 (5.7)	13 (5.7)	0	0.403		
Abdominal cavity	24 (9.3)	22 (9.6)	2 (7.1)	0.937		
Catheter related	2 (0.8)	2 (0.9)	0	1.000		
BSI	32 (12.5)	24 (10.5)	8 (28.6)	0.015*	4.02 (0.18–89.40)	0.379
**Length of hospitalization**	21.0 (12.0–28.0)	21.0 (13.0–28.0)	11.0 (7.0–16.0)	< 0.001*		
≥ 21.0	130 (50.6)	126 (55.0)	4 (14.3)	< 0.001*	0.06 (0.01–0.43)	0.005*
**Empirical antibiotics treatment**	224 (87.2)	198 (86.5)	26 (92.9)	0.512		
β-lactam/β-lactamase inhibitor combinations	41 (16.0)	36 (15.7)	5 (17.9)	0.986		
Cephalosporins	22 (8.6)	20 (8.7)	2 (7.1)	1.000		
Carbapenems	9 (3.5)	9 (3.9)	0	0.601		
Fluoroquinolones	22 (8.6)	19 (8.3)	3 (10.7)	0.941		
Aminoglycosides	5 (1.9)	5 (2.2)	0	0.948		
Combination therapy	125 (48.6)	109 (47.6)	16 (57.1)	0.340		
**Effective empirical antibiotics treatment**	134 (52.1)	118 (51.5)	16 (57.1)	0.575		
**Length of antibiotics treatment (days)**	8.0 (5.0–13.0)	8.0 (5.0–13.0)	6.5 (4.0–9.8)	0.143		
**ICU admission**	33 (12.8)	30 (13.1)	3 (10.7)	0.955		
**Mechanical ventilation**	18 (7.0)	14 (6.1)	4 (14.3)	0.227		
**Septic shock**	38 (14.8)	27 (11.8)	11 (39.3)	< 0.001*	1.27 (0.17–9.48)	0.814
**Laboratory examination results**						
Hemoglobin (g/L; normal range 115–150)	106.0 (93.0–117.0)	107.0 (94.5–118.5)	99.0 (80.8–107.0)	0.001*		
< 110	148 (57.6)	125 (54.6)	23 (82.1)	0.005*	9.15 (1.22–68.66)	0.031*
Platelet count (×10^9^/L; normal range 125–350)	212.0 (139.0–278.0)	219.0 (147.5–281.5)	123.5 (51.0–225.3)	0.009*		
< 100.0	32 (12.5)	21 (9.2)	11 (39.3)	< 0.001*	4.41 (0.61–36.91)	0.142
White-cell count (×10^9^/L; normal range 4.0–10.0)	7.0 (4.8–10.0)	6.9 (4.8–9.8)	7.3 (3.4–11.6)	0.521		
> 10.0	64 (24.9)	55 (24.0)	9 (32.1)	0.348		
< 4.0	46 (17.9)	39 (17.0)	7 (25.0)	0.299		
Neutrophils count (× 10^9^/L; normal range 1.8–6.3)	5.3 (3.1–8.2)	5.3 (3.1–8.0)	6.1 (2.5–10.3)	0.812		
Lymphocytes count (×10^9^/L; normal range 1.1–3.2)	0.9 (0.6–1.3)	0.9 (0.6–1.3)	0.6 (0.3–0.9)	0.014*		
< 1.0	152 (59.1)	129 (56.3)	23 (82.1)	0.009*	2.43 (0.29–20.48)	0.414
PCT (ng/mL; normal range 0–0.5)	0.5 (0.5–0.9)	0.5 (0.5–0.5)	1.2 (0.5–6.7)	0.139		
≥ 1.0	62 (24.1)	46 (20.1)	16 (57.1)	< 0.001*	0.82 (0.14–5.00)	0.833
Albumin (g/L; normal range 40–55)	34.6 (30.0–39.0)	35.0 (30.0–39.0)	31.0 (26.4–34.8)	< 0.001*		
< 30.0	61 (23.7)	49 (21.4)	12 (42.9)	0.012*	3.55 (0.53–23.80)	0.193

*Abbreviations*: *MDR* multidrug-resistant, *OR* odds ratio, *CI* confidence interval, *BSI* bloodstream infection, *ICU* intensive care unit, *PCT* procalcitonin

“*” represents *P* < 0.05

### Comparison of clinical and infection-related characteristics in the study population based on the survival status of patients during hospitalization

We used in-hospital mortality data to evaluate the primary clinical outcomes of nosocomial infections caused by MDR bacteria in cancer patients. Among the study subjects, the overall case-fatality rate was 10.9% (28/257). We also analyzed the relationship between prognosis and clinical characteristics of these infections in cancer patients. The results showed that smoking history, ECOG performance status, existence of distant metastasis, presence of liver disease, CCI, existence of fever, underwent surgery or chemotherapy within 30 days, received intrapleural/abdominal infusion within 30 days, and the presence of indwelling catheters or other devices (indwelling urinary catheters and drains postoperation) significantly differed between survivors and non-survivors (*P* < 0.05; Table 1). Additionally, sample type (sputum and urine), primary sites of infection (respiratory tract infection, urinary tract infection, and BSI), length of hospitalization, septic shock, and laboratory examination results (hemoglobin, platelet count, lymphocytes count, and albumin) also differed between survivors and non-survivors (*P* < 0.05; Table 2).

### Bacterial characteristics

During the six years, there were 257 cultures isolated from different clinical specimens. The majority of specimens were urine (36.2%), followed by sputum (24.1%), blood culture (17.1%), wounds secreta (8.2%), and drainage fluid postoperation (8.2%). The causative pathogens of nosocomial infection episodes were compared by survival status during hospitalization in Table 3. Overall, ESBL-PE members were the most frequently isolated MDR strains (72.8%), followed by *Acinetobacter baumannii* (11.7%), *Stenotrophomonas maltophilia* (6.2%), MDR *Pseudomonas aeruginosa* (5.1%), and carbapenem-resistant Enterobacteriaceae (1.6%). MRSA was the only isolated multidrug-resistant gram-positive bacterium, accounting for 2.7%. However, there was no significant difference in causative pathogen distribution (*P* > 0.05) between the survivor and non-survivor groups (Table 3).

**Table 3:** Causative agents of all nosocomial infection episodes caused by MDR bacteria in cancer patients

Causative organisms	All *N* = 257	Survivor *N* = 229, n (%)	Non-survivor *N* = 28, n (%)	*P*-value
MRSA	7 (2.7)	5 (2.2)	2 (7.1)	0.364
ESBL-PE	187 (72.8)	167 (72.9)	20 (71.4)	0.867
MDR *Pseudomonas aeruginosa*	13 (5.1)	13 (5.7)	0	0.403
*Acinetobacter baumannii*	30 (11.7)	27 (11.8)	3 (10.7)	1.000
*Stenotrophomonas maltophilia*	16 (6.2)	13 (5.7)	3 (10.7)	0.531
Carbapenem-resistant Enterobacteriaceae	4 (1.6)	4 (1.7)	0	1.000

*Abbreviations*: *MDR* multidrug-resistant, *MRSA* Oxacillin-resistant *Staphylococcus aureus*, *ESBL-PE* extended-spectrum β-lactamase-producing Enterobacteriaceae

### Risk factors for in-hospital mortality

In this study, the univariate analysis results demonstrated that smoking history, ECOG performance status, existence of distant metastasis, presence of liver disease, CCI, existence of fever, underwent surgery or chemotherapy within 30 days, received intrapleural/abdominal infusion within 30 days, presence of indwelling catheters or other devices (indwelling urinary catheters and drains postoperation), sample type (sputum and urine), primary sites of infection (respiratory tract infection, urinary tract infection, and BSI), length of hospitalization, septic shock, and laboratory examination results (hemoglobin, platelet count, lymphocytes count, and albumin) were significantly correlated with the in-hospital mortality. The multivariate analysis results identified smoking history, received intrapleural/abdominal infusion within 30 days, presence of indwelling urinary catheters, length of hospitalization, and hemoglobin as independent prognostic factors for in-hospital mortality among cancer patients with nosocomial infections due to MDR bacteria (Tables 1 & 2).

### Antimicrobial susceptibility analysis

**Fig. 2:**
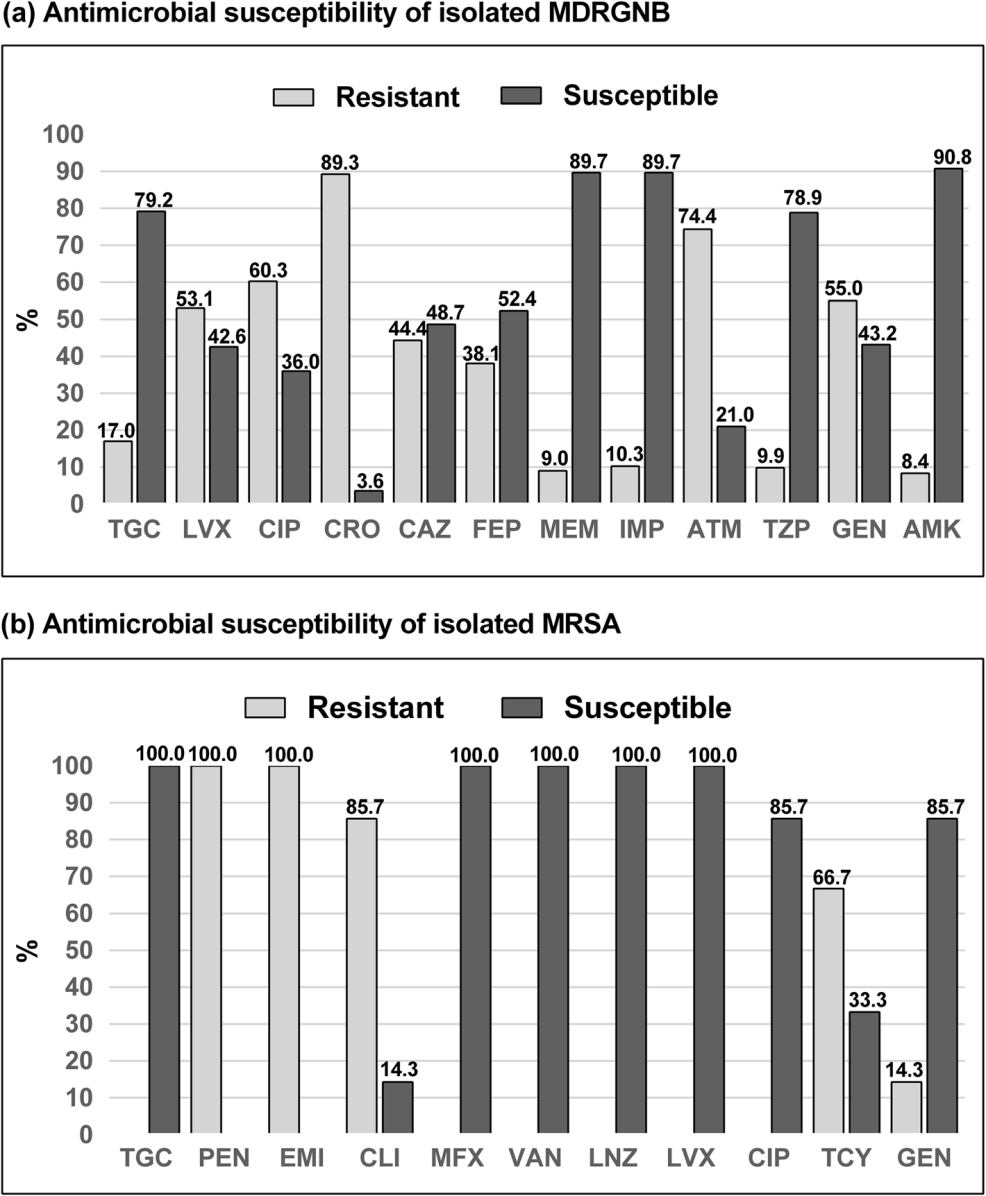
Antimicrobial susceptibility patterns of nosocomial infections caused by MDR bacteria in cancer patients. **a** The isolated MDRGNB. **b** The isolated MRSA. TGC Tigecycline, LVX Levofloxacin, CIP Ciprofloxacin, CRO Ceftriaxone, CAZ Ceftazidime, FEP Cefepime, MEM Meropenem, IMP Imipenem, ATM Aztreonam, TZP Piperacillin/tazobactam, GEN Gentamicin, AMK Amikacin, PEN Penicillin, CLI Clindamycin, MFX Moxifloxacin, TEC Teicoplanin, VAN Vancomycin, LNZ Linezolid, MNO Minocycline, TCY Tetracycline.

The antimicrobial sensitivity of commonly used antibiotics showed that the isolated MDRGNB were primarily sensitive to meropenem, imipenem, and amikacin, while they were primarily resistant to ceftriaxone, aztreonam, and ciprofloxacin (Fig. 2a). MRSA was the only isolated MDR gram-positive bacterium, and the drug sensitivity analysis showed that these strains were primarily sensitive to vancomycin, linezolid, moxifloxacin, levofloxacin, and tigecycline (Fig. 2b).

## Discussion

In this retrospective study, we observed that the prevalence of nosocomial infections caused by MDR bacteria in cancer patients was 25.5%, which was consistent with the results of a recent study conducted in Spain (25.5%) [23]. However, in the Spain study, the subjects were cancer patients with febrile neutropenia (FN), and all of the organisms were isolated from blood culture. At the same time, a prospective observational study conducted in Turkey showed that MDR bacteria isolated from all cultures caused 36.3% of nosocomial infection episodes, while only 17.1% of cases were identified as nosocomial infection episodes if blood cultures were included. This result suggests that we should pay more attention to other sites of infections (such as urinary tract, respiratory tract, and gastrointestinal tract infections) rather than focusing merely on BSIs, even though BSIs are more worrisome and are associated with high mortality.

In this study, we observed that ESBL-PE was the most frequent organism causing nosocomial infection episodes in cancer patients, accounting for 72.8% of such episodes, followed by *Acinetobacter baumannii* (11.7%), and *Stenotrophomonas maltophilia* (6.2%). Compared with gram-negative bacteria, MRSA was the only isolated gram-positive MDR bacterium, accounting for 2.7% of isolates. Biehl et al. [24] reported that ESBL-producing Enterobacteriaceae are emerging as a new threat for both nosocomial and community infections worldwide, and ESBL-PE caused approximately 1 in 10 nosocomial infection episodes in patients with malignancy. At the same time, nosocomial infections caused by ESBL-PE were more worrisome due to the increased mortality in these patients [24]. Therefore, rapid initiation of appropriate and adequate antibiotic therapy is pivotal for nosocomial infection episodes caused by ESBL-PE, since most empirical regimens do not adequately cover these pathogens [1].

Among the study subjects, a considerable overall case-fatality rate of 10.9% was observed in our study, which was lower than that observed in most previous studies conducted in other countries [13, 25, 26]. According to a retrospective conducted in Brazil, Freire et al. reported that the overall case-fatality rate of carbapenem-resistant *K. pneumoniae* caused nosocomial infection reached 57.8% in patients with solid tumors despite its small sample size in this cohort (83 infection episodes) [25]. In a case-control study, including 204 cancer patients admitted to ICU, Nazer et al. reported that the 30-day mortality exceed 70% for cancer patients with nosocomial infections caused by *Acinetobacter baumannii* [26]. At the same time, Moghnieh et al. reported that the case-fatality rates of nosocomial infections caused by MDR bacteria up to 57.1% in cancer patients with FN [27]. Recently, in a five-year period retrospective study that included 73 patients with solid tumors, Perdikouri et al. reported that patients died in 30% due to infections caused by MDR bacteria [13],which may have been due to the majority of patients having been at an advanced stage and had distant metastasis.

The results of the multivariate analysis showed that former smokers were associated with a higher case-fatality rate in cancer patients with MDR bacteria caused nosocomial infections, which was an interesting finding in our study. Stämpfli et al. reported that cigarette exposure significantly impacts the immune system, impairing the ability of a host to produce appropriate immune and inflammatory responses and promoting infection [28]. We observed that cancer patients who received an intrapleural/abdominal infusion within 30 days were associated with a higher case-fatality rate in our cases, possibly suggesting that these patients are more likely to experience an MDR bacterial infection, or catheter-related infection and can be easily immunocompromised [2]. The presence of indwelling urinary catheters was also observed to be an independent risk factor for mortality in cancer patients with nosocomial infections caused by MDR bacteria. This finding is consistent with those of previous studies conducted elsewhere in patients with nosocomial infections caused by MDR [29, 30]. The results of our study demonstrated that prolonged hospitalization was associated with decreased in-hospital mortality in these patients. In contrast, Perdikouri et al. reported that prolonged hospitalization was associated with an increased fatality rate in cancer patients [13]. This discrepancy can be explained by survivorship bias since we used different study outcomes to evaluate the prognosis of infection episodes. Therefore, the results need to be further validated before drawing conclusions. In addition, anemia was also observed to be an independent risk factor for mortality in cancer patients with nosocomial infections caused by MDR bacteria. Zhang et al. reported that pretreatment anemia-induced tissue hypoxia may directly reduce the overall survival of cancer patients [31].

The antimicrobial susceptibility results showed that the isolated MDRGNB were primarily sensitive to meropenem, imipenem, and amikacin, while they were primarily resistant to aztreonam, cephalosporins (third or fourth generation), and fluoroquinolone. MRSA was the only isolated MDR gram-positive bacterium, and the drug sensitivity analysis results showed that these strains were primarily sensitive to vancomycin, linezolid, moxifloxacin, levofloxacin, and tigecycline, which was comparable with the results of previous studies [32, 33]. The phenomenon of MDR can be attributed to the overuse of antibiotics in China. In recent years, a growing number of studies suggest that efflux pumps play an essential role in the mechanism of MDR [34]. The overexpression of multidrug efflux pumps is closely associated with drug resistance, where these pumps can expel a broad range of antibiotics, decreasing the antibiotic concentration in the cell and promoting mutation accumulation [35]. Previous studies also revealed that the overexpression of the efflux pumps AcrAB, MexAB-OprM and OprD was correlated with high-level fluoroquinolone and carbapenem resistance [35, 36]. Thus, there is an urgent need to detect mutations known to cause the overexpression of efflux pumps to allow for the precise management of nosocomial infections caused by MDR bacteria in cancer patients. In addition, the entire microbial spectrum should be taken into consideration when initiating empirical antibiotic treatment [32]. To the best of our knowledge, this is the first study to evaluate the clinical characteristics, microbial spectrum, antibiotic resistance patterns, and prognostic factors among cancer patients with nosocomial infections caused by MDR bacteria in China, and one of the advantages of our study is that we studied large number of risk factors.

Our study has several limitations. First, it was difficult to collect some variables (e.g., concrete chemotherapeutic or radiation dosage, concrete antibiotics treatment before admission, and some laboratory examination results such as C-reactive protein, (1,3)-β-D-glucan test, and galactomannan test) in this retrospective study. Thus, there may be hidden biases in the analysis of the relationships. In addition, although a portion of MDR infection episodes were identified by the qualified clinician based on the clinical manifestations of the patients in the medical records, this method may have hidden selection and information biases and have poor sensitivity and specificity. Furthermore, our study was a single-center retrospective study, and a prospective multicenter study is needed for further validation.

## Conclusions

In summary, nosocomial infections due to MDR bacteria in oncological patients were associated with higher prevalence and in-hospital mortality in our study. The most frequently isolated pathogens were ESBL-PE, *Acinetobacter baumannii*, and *Stenotrophomonas maltophilia*. The isolated MDR bacteria exhibited high sensitivity to meropenem, imipenem, and amikacin. Former smokers, intrapleural/abdominal infusion history within 30 days, presence of indwelling urinary catheters, and anemia were independent risk factors for in-hospital mortality of nosocomial infections caused by MDR bacteria. We suggest that clinicians should focus more on nosocomial infections caused by MDR in cancer patients, and consider the epidemiological characteristics of local resistance patterns when initiating antimicrobial treatment.

## Data Availability

Please contact author for data requests.

## Abbreviations

MDR: Multidrug-resistant
ESBL-PE: Extended-spectrum β-lactamase-producing Enterobacteriaceae
MDRGNB: Multidrug-resistant Gram-negative bacteria
CRE: Carbapenem-resistant Enterobacteriaceae
MRSA: Methicillin-resistant *Staphylococcus aureus*
FN: Febrile neutropenia
OR: Odds ratio
CI: Confidence interval
ECOG: Eastern Cooperative Oncology Group
AJCC: American Joint Committee on Cancer
CCI: Charlson comorbidity index
G-CSF: Granulocyte colony-stimulating factor
ICU: Intensive care unit
PCT: Procalcitonin
CVC: Central venous catheter
PICC: Peripherally inserted central catheter
TGC: Tigecycline
LVX: Levofloxacin
CIP: Ciprofloxacin
CRO: Ceftriaxone
CAZ: Ceftazidime
FEP: Cefepime
MEM: Meropenem
IMP: Imipenem
ATM: Aztreonam
TZP: Piperacillin/tazobactam
GEN: Gentamicin
AMK: Amikacin
PEN: Penicillin
CLI: Clindamycin
MFX: Moxifloxacin
TEC: Teicoplanin
VAN: Vancomycin
LNZ: Linezolid
MNO: Minocycline
TCY: Tetracycline
